# Prenatal Attachment and Mental Well-Being Among Expectant Fathers Amidst COVID-19: A Cross-Sectional Study

**DOI:** 10.7759/cureus.44806

**Published:** 2023-09-06

**Authors:** Berhan Akdağ, Dilek Erdem, Funda İpekten, Emre Han Alpay, Figen Yardımcı, Murat Bektaş

**Affiliations:** 1 Child and Adolescent Psychiatry, Silifke State Hospital, Mersin, TUR; 2 Department of Gynecology and Obstetrics, Alaaddin Keykubat University Alanya Training and Research Hospital, Antalya, TUR; 3 Department of Biostatistics and Medical Informatics, Adıyaman University School of Medicine, Adıyaman, TUR; 4 Department of Psychology, Mersin University, Mersin, TUR; 5 Department of Pediatric Nursing, Ege University, İzmir, TUR; 6 Department of Pediatric Nursing, Dokuz Eylül University, İzmir, TUR

**Keywords:** well-being, risk perception, prenatal attachment, expectant father, covid-19

## Abstract

Background

The COVID-19 pandemic has posed significant threats to global physical and mental health, notably impacting the psychological management of pregnancy. The mental health of parents plays a critical role in fostering the emotional bond with their unborn child, referred to as prenatal attachment. Despite the significance of this bond, research has primarily concentrated on maternal outcomes, often neglecting the paternal aspect during the pandemic. This study investigates the correlation between coronavirus disease 2019 (COVID-19) risk perception and paternal prenatal attachment, further exploring the mediating role of well-being within this association.

Methods

A total of 141 expectant fathers attending the gynecology and obstetrics outpatient clinic with their partners were recruited. Participants completed measures including the Paternal Antenatal Attachment Scale (PAAS), the World Health Organization Well-being Index (WHO-5), and the COVID-19 Perceived Risk Scale (CPRS).

Results

Data analysis revealed a significant negative correlation between COVID-19 risk perception and well-being (ß = -.34, *p* < .001). There was a positive correlation between well-being and prenatal attachment (ß = .37, *p* = .002). The prenatal attachment was also positively linked to COVID-19 risk perception (ß = .20, *p* = .047). Furthermore, well-being mediated the relationship between COVID-19 risk perception and prenatal attachment.

Conclusion

The findings underscore the potential of the COVID-19 risk perception to disrupt the prenatal attachment process for expectant fathers by interfering with psychological well-being. However, it can also promote prenatal attachment through various mechanisms. Consequently, acknowledging and understanding the experiences of fathers during pregnancy is of paramount importance. Future longitudinal studies are necessitated to examine the parent-child relationship dynamics that have evolved under the influence of the pandemic.

## Introduction

The literature defines "attachment" as the connection between a child and their caregiver [1]. This bond is fundamental for the child's ability to explore the world and seek comfort and safety during challenging moments. As Bowlby's attachment theory suggests, a child establishes a distinctive attachment style with their caregivers within the first year of their life [1]. This attachment style can either be "secure" or "insecure," depending on the child's early relational experiences [2]. When the child receives kindness and care from their caregivers, they develop a "secure" attachment. This is a vital aspect, as a secure bond established early in life can have a positive impact on long-term mental health. Securely attached children tend to be more resilient, adaptable, and self-assured. Additionally, they experience better relationships with others during adulthood [3]. Consequently, parenting attachment is crucial in promoting the child's healthy development.

The concept of “prenatal attachment”

The emotional connection between parents and their offspring often begins prenatally when the child is still in utero [4]. Researchers have adopted the term "attachment" to describe the emotional bond that parents form with their unborn baby, and this bond has been conceptualized as “prenatal attachment” [5]. Prenatal attachment plays a vital role in preparing parents mentally for impending parenthood [5]. It also significantly impacts the child’s psychosocial and emotional development, the quality of parental care, and the future parent-child relationship [6]. Indeed, parents who feel deeply attached to their unborn baby are more likely to provide a nurturing and secure environment for their child's healthy development.

Unsurprisingly, previous studies addressing the parent-child connection have focused on mothers [4]. However, nowadays, with women becoming more actively involved in professional life and the predominance of nuclear family systems, the role of fathers in child-rearing has expanded [7]. This underscores the necessity to comprehensively understand the attachment experiences of expectant fathers during the prenatal period. From a male perspective, prenatal attachment is instrumental in fostering the development of a “paternal” identity [6]. This is critical because fathers who actively and regularly engage with their children have a significant impact on all aspects of children’s development later in life [8]. Furthermore, it has been associated with positive pregnancy outcomes. Men who exhibit higher levels of prenatal attachment tend to demonstrate a heightened sensitivity to their partner’s physical and emotional needs throughout pregnancy [6].

The strength of prenatal attachment may fluctuate across the span of pregnancy. Accumulating evidence indicates that prenatal attachment intensifies throughout gestation, reaching its zenith in the final trimester [3]. Factors such as younger parental age, a deliberate intention for the pregnancy, and higher educational attainment have been linked to greater emotional investment in the unborn child [9,10]. However, mental health issues (e.g., anxiety and depression) can impede the formation of a strong attachment to the unborn child. For parents who experience poor psychological well-being, it can be challenging to form an emotional connection with their unborn baby [11,12]. Several factors can also contribute to this, including the dearth of emotional resources, self-perceived parenting inadequacies, and negative attitudes toward caregiving responsibilities [13].

The rationale of the present study

The ongoing COVID-19 pandemic has dramatically reshaped societal norms and introduced unprecedented stressors, such as job loss and the potential death of self or loved ones. Pregnant women have been uniquely susceptible to adverse mental health outcomes due to an amplified risk of severe COVID-19 illness, preterm labor, and additional pregnancy complications [14]. Consequently, the pandemic period saw a surge in mental health disorders among this population [15].

Studies focusing on prenatal attachment in pregnant women revealed an inverse correlation between pandemic-induced mental health issues and prenatal attachment. For instance, Albayrak and colleagues [16] found that pregnant women who had low levels of anxiety and obsession related to COVID-19 had significantly higher prenatal attachment scores than those who had high levels of anxiety and obsession. Similarly, Karaca and colleagues [17] found a negative and significant relationship between coronavirus anxiety and prenatal attachment scores among pregnant women who were more than 20 weeks into their pregnancy. Craig and colleagues also revealed an adverse relationship between state anxiety and prenatal attachment; however, this relationship was not observed in pregnant women who have a higher COVID-19 risk perception [18]. Nevertheless, there remains a dearth of literature examining the relationship between psychological well-being and prenatal attachment in the context of expectant fathers during the pandemic. To bridge this knowledge gap, the present study seeks to probe the relationship between paternal perception of COVID-19 risk and prenatal attachment, with a particular focus on the possible mediating role of psychological well-being in this relationship. Accordingly, we propose the following hypotheses: H1 - There is a negative correlation between COVID-19 risk perception and well-being; H2 - There is a negative correlation between COVID-19 risk perception and paternal prenatal attachment; H3 - There is a positive correlation between well-being and paternal prenatal attachment; H4 - Well-being serves as a mediating factor in the relationship between COVID-19 risk perception and paternal prenatal attachment.

## Materials and methods

Participants and study procedure

This research, conducted from November to December 2021, employed a descriptive and cross-sectional study design at the Gynecology and Obstetrics Outpatient Clinic of Alanya Training and Research Hospital. Alanya is a city located on the Mediterranean coast of Turkey, with a population of around 400,000 inhabitants. The hospital is the biggest in the area and is free of charge. Permission to conduct this study was obtained from the Alaaddin Keykubat University (ALKU) Health Sciences Scientific Research and Publication Ethics Committee (Approval no: 2021/17-06).

For the current study, a convenience sampling technique was utilized. Expectant fathers accompanying their partners to the clinic were invited to participate in the study; 141 of them agreed to participate in the study. Thus, the participant cohort consisted of expectant fathers (n = 141) accompanying their partners to the clinic. Inclusion criteria for the study included: a) the ability to fluently speak and understand Turkish, b) a minimum age of 18 years, and c) the absence of health issues that could potentially interfere with the study, such as intellectual disabilities or mental health conditions (e.g., psychosis and bipolar disorder). Prior to participation, all candidates were thoroughly briefed about the objectives of the study, after which their informed consent was obtained to proceed with the questionnaire completion.

Measures

The Paternal Antenatal Attachment Scale (PAAS) is a 16-item instrument employed to measure antenatal attachment among expectant fathers [19]. This instrument incorporates two distinct subscales: quality of attachment, which refers to the emotional response upon contemplation of the unborn child, and time spent in attachment mode, a metric representing the intensity of involvement with the unborn child. Items are assigned scores ranging from 1 to 5, leading to an overall possible score range from 16 to 80. Both subscales and the total attachment score can be calculated independently, with higher scores indicative of elevated levels of paternal prenatal attachment [20]. Our study utilized the total attachment score.

The WHO Well-being Index (WHO-5) serves as an efficient tool for mental well-being screening [21]. Constructed as a unidimensional scale, this tool boasts strong construct validity in the measurement of well-being on a 5-point Likert scale (0 = never and 5 = always) about the past two weeks. Higher scores correlate to greater well-being. The Turkish version’s validity and reliability were established by Eser et al., recording a Cronbach’s alpha of 0.81 [22].

The COVID-19 Perceived Risk Scale (CPRS) is an 8-item tool utilized to gauge COVID-19 risk perception. It encompasses two dimensions of risk perception: cognitive and emotional. Each item receives a score from 1 (negligible) to 5 (very large), resulting in a total potential score range from 8 to 40. Higher scores signify an increased perception of COVID-19 risk. The tool’s initial development study revealed satisfactory internal consistency within the Turkish population [23].

Statistical analysis

To ascertain the required sample size, a power analysis was conducted based on a medium effect size (f2 = 0.15), a statistical significance level of 0.05, and a target power of 0.95. The analysis indicated that a minimum sample size of 107 was required. Descriptive analyses were then performed on the variables using SPSS 28.0 statistical software (IBM Corp., Armonk, NY). The data's normality was assessed through skewness/kurtosis values, box plots, and histograms. Pearson's correlation analysis was utilized to examine the relationships between the variables. Additionally, Student's t-test and one-way ANOVA were employed to determine any differences in attachment scores between groups.

Our final step involved the construction of a mediation model (model 4), executed using the PROCESS macro for SPSS [24]. This model investigated the relationship between COVID-19 risk perception and paternal prenatal attachment, along with the potential mediating role of well-being in this relationship. We used non-parametric bootstrapping with 10,000 samples to test the indirect effects. The indirect effect was deemed significant if zero was not included in the 95% confidence intervals (CIs).

## Results

Table 1 summarizes the sociodemographic characteristics of the study participants. 

**Table 1:** Sociodemographic characteristics of the participants (n = 141) COVID-19: Coronavirus disease 2019

	M (SD)
Age (years)	32.41 (6.25)
	n (%)
Educational level	
Illiterate or primary school	26 (18.4)
Secondary school	34 (24.1)
High school	54 (38.3)
University or above	27 (19.1)
Presence of chronic disease	
No	134 (95.0)
Yes	7 (5.0)
COVID-19 vaccine status	
Complete	94 (66.7)
Incomplete or not vaccinated	47 (33.3)
History of confirmed COVID-19	
No	112 (79.4)
Yes	29 (20.6)
Loss of relatives due to COVID-19	
No	116 (82.3)
Yes	25 (17.7)
Income changes during the pandemic	
Decreased	106 (75.1)
Not change	29 (20.6)
Increased	6 (4.3)
Trimester	
First or Second	42 (29.8)
Third	99 (70.2)
Baby expecting status	
First time	59 (41.8)
Subsequent	82 (58.2)
History of abortus or stillbirth	
No	107 (75.9)
Yes	34 (24.1)
High-risk current pregnancy	
No	128 (90.8)
Yes	13 (9.2)
Planned current pregnancy	
No	34 (24.1)
Yes	107 (75.9)

Table 2 presents the means, standard deviations, and inter-correlations. We observed a significant inverse correlation between COVID-19 risk perception and well-being (r = -.414, p < .001). Furthermore, a statistically significant positive association was found between well-being and paternal prenatal attachment (r = .204, p = .015).

**Table 2:** The correlations among variables *p<0.05, **p<0.01, ***p<.001, Pearson correlation analysis, Kurt: Kurtosis, M: Mean, SD: Standard deviation, Skew: Skewness COVID-19: Coronavirus disease 2019

Variables		1	2	3	M	SD	Skew.	Kurt.
COVID-19 Risk Perception		-			21.30	6.87	.282	.407
Well-being		-.417***	-		14.70	5.67	-.102	-.708
Paternal Prenatal Attachment		-.065	.204*	-	34.30	3.99	-.356	-.457

Our study demonstrated that first-time expectant fathers scored significantly higher on the PAAS compared to those with previous children (p < .001). Likewise, participants expecting a planned baby also exhibited significantly elevated PAAS scores relative to their counterparts (p = .034). These findings are detailed in Table 3.

**Table 3:** Differences in the PAAS scores between the groups ^a^Student’s t-test, PAAS: Paternal antenatal attachment scale, Mean (Standard deviation). Note: All variables underwent analysis, but only the statistically significant findings were shown in the table.

	PAAS scores	p-value
Planned current pregnancy		
No	61.3 (8.77)	0.034^a^
Yes	64.4 (6.92)	
Baby expecting status		
First time	65.6 (7.51)	<.001^a^
Subsequent	62.3 (7.22)	

Mediation analyses

**Figure 1:**
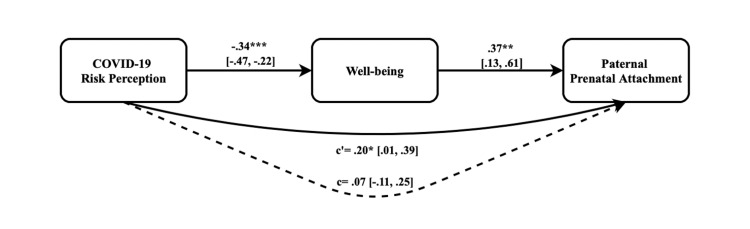
Conceptual and statistical diagram *p<0.05, **p<0.01, ***p<.001, c'= direct effect, c= total effect. All pathways are unstandardized, and a pathway that is not statistically significant is indicated by the dashed line. COVID-19: Coronavirus disease 2019

To examine the interrelation between COVID-19 risk perception, paternal prenatal attachment, and the potential intermediary role of well-being, a mediation analysis was conducted. The results of this analysis are visually represented in Figure 1.

A significant negative correlation was found between high COVID-19 risk perception and well-being among expectant fathers (ß = -.34, 95% CI (-.22, -.47), p < .001). Concurrently, a significant positive correlation was identified between well-being and paternal prenatal attachment (ß = .37, 95% CI (.13, .61), p = .002).

The direct effect of COVID-19 risk perception on paternal prenatal attachment was significant (ß = .20, 95% CI (.01, .39), p = .047). However, the total effect did not reach statistical significance (ß = .07, 95% CI (-.11, .25), p = .447). Notwithstanding, the path from COVID-19 risk perception to paternal prenatal attachment, mediated by well-being, yielded statistically significant results (ß = -.13, 95% CI (-.24, -.04)).

## Discussion

While previous research has thoroughly explored the correlation between maternal mental health issues and prenatal attachment during pandemic times, such studies have not examined this relationship within a cohort of expecting fathers. Our present study aimed to address this gap by examining the relationship between paternal psychological well-being and prenatal attachment, integrating elements of COVID-19 risk perception. 

Our findings indicated a positive correlation between paternal psychological well-being and prenatal attachment, aligning with existing literature on the topic. For instance, Vreeswijk and colleagues [7] found that expecting fathers reported enhanced attachment quality if they experienced fewer symptoms of depression and anxiety during the pregnancy period, as evidenced by a Dutch community-based sample. Meanwhile, Brandão and colleagues [25] demonstrated that depressive symptoms in non-first-time fathers correlated with poorer prenatal attachment, specifically when these fathers experienced low levels of positive dyadic coping. Contrastingly, first-time fathers showed a consistent association between depressive symptoms and poorer prenatal attachment, irrespective of their positive dyadic coping levels. It is noteworthy that becoming a parent necessitates psychological adaptation and effort, and mental health disturbances during pregnancy could impede this transition. The extent of focus on attachment to the unborn child could also fluctuate based on the parents’ psychological well-being. Consequently, expecting fathers dealing with mental health issues during the prenatal period might encounter challenges in conceptualizing their unborn child. 

Our research findings demonstrate an inverse relationship between COVID-19 risk perception and overall well-being. Risk perception reflects an individual’s subjective assessment of the potential severity and characteristics of a given risk, influenced by a myriad of affective, cognitive, contextual, and individual factors [26]. There is a strong correlation between risk perception and mental health, with prolonged exposure to perceived threatening situations potentially leading to mental health issues [27]. Nevertheless, this relationship is bidirectional. In other words, a person's emotional state can also impact their risk perception, and negative emotions may lead to a more pessimistic view of risk [28]. 

Interestingly, our study reveals a positive correlation between COVID-19 risk perception and paternal prenatal attachment. This relationship may be explained by several possible mechanisms. First, high-risk perception can initiate coping strategies to manage stressful situations. Pregnant women with a heightened perception of risk may cultivate prenatal attachment as an emotional coping strategy to navigate the pandemic [18]. It is well documented that adverse emotions can be mitigated through various methods, such as resolving their root causes, avoiding their inception, or replacing them with neutral or even pleasant emotions [29]. In this regard, the third strategy might shed light on the enhanced prenatal attachment observed among expectant fathers. They may perceive moments contemplating their unborn child as a sanctuary. Second, risk perception is not always detrimental. It can heighten alertness, enhance awareness of potential dangers, and promote health-conscious behavior [30]. As such, expectant fathers with increased COVID-19 risk perception may choose to spend more time at home, allowing them to engage more deeply with their partners and the unborn child.

Pregnancy represents a significant period of change and challenge with profound implications for expectant parents. The magnitude of these changes may become particularly overwhelming in the case of unplanned pregnancies, as parents may have had insufficient time to mentally prepare for the upcoming transformations. Consequently, this lack of preparation can lead to a diminished prenatal attachment for parents faced with an unplanned pregnancy [9,10]. Nevertheless, previous research [7] revealed that first-time expectant fathers often exhibit increased preoccupation and enhanced attachment toward their unborn child in comparison to those who have previously fathered children. In accordance with these established patterns, our study revealed that first-time fathers anticipating a planned child yielded higher scores on the PAAS compared to their counterparts.

This study holds substantial value as it broadens our understanding of the complex relationship between prenatal attachment and psychological well-being. To the best of our knowledge, it is the first study to explore paternal prenatal attachment in the context of a global pandemic. Nonetheless, it is crucial to acknowledge several limitations. Primarily, due to the cross-sectional nature of this study, it was not possible to assess longitudinal relationships between the variables. Secondly, the relatively small sample size may limit the generalizability of the results. Thirdly, a diagnostic interview was not conducted with the fathers. Fourthly, maternal prenatal attachment, which may affect paternal attachment, was not addressed. Lastly, the participant pool consisted solely of expectant fathers who accompanied their partners to prenatal visits. Consequently, it is plausible that this specific group may inherently display higher prenatal attachment scores and lower perception of COVID-19 risk than the general population of expectant fathers.

## Conclusions

The present study conducted on a cohort of expectant fathers has demonstrated a significant correlation between indices of mental health, particularly well-being, and prenatal attachment. It is crucial to note that expectant fathers, like their female counterparts, may encounter mental health issues during pregnancy. However, these issues are often underreported and inadequately addressed by healthcare professionals. The lack of systemic policies providing support for expectant fathers only exacerbates these challenges, potentially escalating feelings of uncertainty and isolation. Consequently, this could have negative implications for the attachment process of expectant fathers. Considering the profound impact of prenatal attachment on the child's psychosocial development and the subsequent parent-child relationship, we propose the need for longitudinal studies. These would enable a thorough evaluation of the “children of the pandemic” in this context, thereby providing valuable insights into mitigating potential future challenges.
